# Progress in Surgery

**Published:** 1898-12-31

**Authors:** 


### Progress in Surgery.

### RHINOLOGY.

**Atrophic Rhinitis**—*Bacteriology*: The presence of a bacillus (?) indistinguishable from the Klebs-Löffler bacillus, in cases of atrophic rhinitis, has induced several practitioners to rely on anti-diphtheritic serum as a method of treatment. We may, however, recall the results recently obtained by Auché and Brindel[1] from the examination of 20 cases. (1) In every case in the course of evolution, with or without ozæna, the diplo-bacillus of Loewenberg was demonstrated, but it was not found in old atrophic coryzas which had apparently been cured. It is not the pathogenic agent of ozæna; (2) The pseudo-diphtheritic bacillus was found in 18 of the 20 cases. It is not the causative agent of ozæna, but only a saprophyte developed in the nasal passages on account of changes in the secretions of mucosa; (3) the bacillus of Pes-Gradenigo was only found in cases of ozæna (with crusts), and this in three only of the 20 cases.

Hecht[2] reports two cases of ozæna treated with electrolysis. Both received six applications, lasting for ten minutes, of from 25 to 30 milliampères. Treatment lasted for two months, and, in addition, the nasal douche was used thrice daily. The first case showed transient improvement. Some time after it was in *statu quo*. In the second case, where he describes the anterior part of the nose as almost normal, there was improvement two months after treatment was finished. The fœtor had subsided, but there was still semifluid greenish secretion present, which was in parts dried into crusts, and could be removed with forceps. He ascribes the want of improvement in the first case to the irreparable atrophy of the mucous membrane. He considers the two cases to support the tropho-neurotic theory of Rethi from a clinical therapeutic basis, and to be against a bacterial origin for ozæna.

*Treatment.*—Dr. George L. Richards[3] speaks highly of formaldehyde in atrophic rhinitis. After removal, by means of a syringe and cotton applicators, of all the crusts and *débris* with a weak alkaline solution, each nostril is thoroughly washed out with a solution of formaldehyde, containing five to ten drops of the 40 per cent. solution to 8 oz. of warm water. It is very irritating even in dilute solutions, and a preliminary spraying of the nose with cocaine is advisable. It produces a temporary sense of smarting. One drop is added to the solution, which the patient uses in the douche-cup for the daily cleansing. Under its use the crusts diminish in number and all unpleasant odour ceases.

**Membranous Rhinitis.**—Lake[4] records a case in a man aged 54, with a right-sided nasal obstruction. He had chronic rhinitis, with unusual pallor of the mucosa, and a number of whitish flakes. He got better, but returned ten months later, saying that his old trouble had recurred. At this time, instead of the whitish flakes, a whitish gelatinous mass, filling entirely the cleft between the septum and the inferior turbinated bone, was found, resembling the "white" of a plover's egg on removal. Both on section and by culture, only staphylococcus pyogenes aureus were found pure. Lake refers to Ball's statement in the last edition of his work, that "the staphylococcus pyogenes aureus in some instances, and the streptococcus pyogenes in others, have been the exciting agents in these cases." Middlemass Hunt[5] defines fibrinous rhinitis as an acute inflammation of the nasal mucous membrane, which is characterised by the presence of false membrane and the absence of any constitutional disturbance, glandular enlargement, albuminuria, or paralysis. He gives notes of four cases, three of which were directly associated with diphtheria, viz.: (1) A medical man, who for two weeks had suffered from membranous rhinitis affecting both nasal passages, and unaccompanied by any constitutional symptoms, developed pharyngeal diphtheria, followed by paralysis, in the third week of the nasal trouble. (2) A little girl, with membrane in both nasal passages, but otherwise in good health, was found to have had pharyngeal diphtheria, with paralysis, six weeks before. Streptococci and staphylococci were found in the membrane, but no Löffler bacilli. (3) A girl, aged 10, had left-sided nasal obstruction, due to the presence of false membrane, and lasting for eight weeks. During that time she was not ill in herself, and had no albuminuria, no enlarged glands, no sore throat, and no paralysis, but a servant in the same house had an attack of "tonsillitis," followed by paralysis of the palate, and a baby died of croup. He summarises our present position in regard to fibrinous rhinitis thus: (1) While admitting that other bacteria besides the Löffler bacillus may give rise to membranous exudation in the nasal passages, the vast majority of cases of fibrinous rhinitis are due to the Löffler bacillus. (2) It is impossible on clinical grounds alone to distinguish fibrinous rhinitis from mild nasal diphtheria. (3) All cases of fibrinous rhinitis should be regarded as diphtheria until the contrary has been proved by reliable bacteriological investigation.

At the Royal Medico-Chirurgical Society Lambert Lack introduced the subject of fibrinous rhinitis for discussion, with special reference to its relation to diphtheria. Fibrinous rhinitis, first described by Schüller in 1871, was defined as a sub-acute or chronic affection of the nose, characterised by a fibrinous or membranous exudation in the nasal mucous membrane. Lack stated that the disease was so common that they formed 2½ per cent. of all the children attending his hospital practice. He gave a brief analysis of 36 cases. The disease was shown to be essentially one of children, and to occur most frequently in the autumn months. The chief symptoms are purely local, such as nasal obstruction and discharge, excoriation of the nostrils and upper lip, occasional epistaxis, &c.; sometimes sore throat. There is complete absence of paralytic sequelæ. The results of bacteriological examination in 33 cases showed that the true Klebs-Löffler bacillus was constantly present, generally in pure culture, sometimes mixed with pyogenic cocci, sarcinæ, &c. It was generally of the large variety, and its identity was proved by its morphology, by its growth on various culture media, &c. It was further shown to be of full virulence on animals, to produce virulent toxins, and to be neutralised by anti-toxins. Inquiry into the surroundings and examination, both clinical and bacteriological, of all persons with whom the patients had come into contact revealed a previous history of diphtheria in connection with one case only, but the disease was very infectious; often it gave rise to itself (nine cases occurred in four families), and often to mild sore throat, 25 instances of which occurred in 11 families. The Klebs-Löffler bacillus was also found in the throats of healthy persons who had been associated with these patients. The conclusion arrived at was that fibrinous rhinitis is a mild form of diphtheria.

T. D. Lister, in the course of discussion, said that he had examined the nasal mucus of 125 children at the East London Hospital for Children. Of these 69 had some nasal discharge, water, mucopurulent or hæmorrhagic. These hæmorrhagic cases all gave pure cultures of the Klebs-Löffler bacillus; and 56 were free from discharge. Of the 69, there were 37 children in whom the Klebs-Löffler bacillus was found, and of the 56 who were free from any nasal discharge whatever no less than 24 presented the bacillus. In no case did ordinary diphtheria arise from these cases coming in contact with others. E W. Goodall said it was well known the Klebs-Löffler bacillus was found in very different conditions, as, *e.g.*, in the sore throat of scarlet fever and in healthy people, but the evidence of a causal connexion between this bacillus and diphtheria was too strong to be given up. He thought that when membrane was confined to the larynx or the nose, constitutional conditions were less severe, possibly because absorption of the toxins was less easy from those surfaces.

**Nasal Hydrorrhœa.**—St. Clair Thomson,[7] in reminding us that attention was first drawn to this subject by Bosworth in 1889, points out that many of Bosworth's cases were not true nasal hydrorrhœa. He had been led to the view that several were cases of escape of cerebro-spinal fluid by the investigation of a case where, in an otherwise perfectly healthy subject, cerebro-spinal fluid with rare intermissions escapes day and night from one side of the nose. This case, together with the records by other observers of eight undoubtedly similar cases, and twelve cases which were most probably identical, will be found fully recorded elsewhere[8]; cases which he terms cerebro-spinal rhinorrhœa. He comes to the conclusion that only three of Bosworth's cases were true nasal hydrorrhœa; one of these is reported by Foster,[9] two were observed by Bosworth himself. Other cases since then have been recorded by Fink[10] (three); Chappell[11] (four); Penyköry[12]; and Jenkelevitch.[13] Thomson thinks that if we exclude from the term nasal hydrorrhœa all cases in which the discharge simply traversed the nose on its way to other cavities, and all those in which it is due to direct or reflex irritation, we find that what has been regarded as a distinct morbid entity is but a symptom of various affections. But he thinks we may still preserve the term "nasal hydrorrhœa" if we limit it by defining the affection as one in which there is a profuse watery discharge secreted by the nasal mucosa, and not depending on any visible intranasal or neighbouring source of irritation. The amount of fluid may vary from what the patient would only term a slight running, up to as much as a pint in the twenty-four hours. Professor Halliburton reported on some of the fluid as follows: "Fluid is thick and viscid and slightly opalescent. On microscopic examination it showed the usual appearances presented by mucus, namely, amorphous matter with mucus corpuscles. It gives with acetic acid, and also with alcohol, a stringy precipitate like that given by mucin. On boiling this precipitate with dilute sulphuric acid, a reducing sugar-like material is formed; this also is characteristic of mucin. The fluid contains a small amount of proteid coagulable by heat; it does not reduce Fehling's solution. Proteoses and peptone are absent. The alcohol extract of the fluid contains no reducing substance. Analysis gives the following results:—

| | | |
|---|---|---|
| Water ... ... ... ... | 98·792 | per 100 |
| Total solids ... ... ... ... | 1·208 | |
| Proteids (including the mucin) ... | 0·260 | |
| Other organic substances ... | 0·163 | |
| Inorganic substances ... .... | 0·785 | |

The presence of mucin and absence of reducing substance, as well as the percentage of proteids and solids, are quite sufficient to distinguish this fluid from normal cerebro-spinal fluid."

Thomson states that in the above case only a few ounces were secreted in the day. In cases where the discharge amounts to a pint or more the fluid is less viscid, and much weaker in mucin. In order to assist other observers in the differential diagnosis of watery discharge from the nose, he has drawn up, after considerable work with Professor Halliburton, the following table of chemical tests for the detection of cerebro-spinal rhinorrhœa: (1) The fluid is perfectly transparent like water, and contains no sediment. (2) It is faintly alkaline in reaction, and either tasteless or slightly salt. (3) The specific gravity is between 1,005 and 1,010. (4) It is not viscous, and gives no precipitate (mucin) on adding acetic acid. (5) On boiling there is not more than a trace of coagulum of serum globulin and serum albumin. (6) Cold nitric acid gives a precipitate, which disappears on heating, and separates again on cooling. (7) Saturation with magnesium sulphate should give a precipitate. Saturation with sodium chloride should also produce a precipitate. Ammonium sulphate should be tried if the above salts fail. (8) The liquid should give a pink or rose-red colour with a trace of copper sulphate and excess of caustic potash, (9) When boiled with Fehling's solution there should be a reduction of the copper (due to pyrocatechin, or some similar body). (10) The reducing substance may be obtained by evaporating to dryness an alcoholic extract of the fluid. It is then found in the form of needle-like crystals. (11) The aqueous solution of this residue does not ferment with yeast.

If applied to suspected cases these tests will in future avoid any question as to the true nature of cerebro-spinal fluid when it escapes from the nose.

1 Rev. Hebd. de Lar., No. 41, 1897. 2 Jour. of Laryng., Oct., 1898; Münch. Med. Woch, 1898, No. 7. 3 New York Med. Jour., Vol. LXVII, p. 826; Amer. Med. Surg. Bull, July 10, 1898. 4 Laryngoscope, Sept., 1898. 5 Rep. of Meeting of B.M.A. at Edinburgh, Brit. Med. Jour., Oct, 22, 1898. 6 Lancet, Oct. 29, 1898. 7 Brit. Med. Jour., Oct. 22, 1898. 8 Med. Chir. Soc. Trans., 1898, Vol. LXXXII. 9 New York Med. Times, 1852, Vol. II., pp. 113-115. 10 Inter. Cent. fur Lar., 1896, No. 9 11 New York Med. Jour., Sept. 29, 1894. 12 Int. Klin. Rundschau, Nov. 24, 1889. 13 Res. sur les Kyotes Muqueux du Sunis Max, Paris, 1860.

